# Assessment of Response to Lithium Maintenance Treatment in Bipolar Disorder: A Consortium on Lithium Genetics (ConLiGen) Report

**DOI:** 10.1371/journal.pone.0065636

**Published:** 2013-06-19

**Authors:** Mirko Manchia, Mazda Adli, Nirmala Akula, Raffaella Ardau, Jean-Michel Aubry, Lena Backlund, Claudio EM. Banzato, Bernhard T. Baune, Frank Bellivier, Susanne Bengesser, Joanna M. Biernacka, Clara Brichant-Petitjean, Elise Bui, Cynthia V. Calkin, Andrew Tai Ann Cheng, Caterina Chillotti, Sven Cichon, Scott Clark, Piotr M. Czerski, Clarissa Dantas, Maria Del Zompo, J. Raymond DePaulo, Sevilla D. Detera-Wadleigh, Bruno Etain, Peter Falkai, Louise Frisén, Mark A. Frye, Jan Fullerton, Sébastien Gard, Julie Garnham, Fernando S. Goes, Paul Grof, Oliver Gruber, Ryota Hashimoto, Joanna Hauser, Urs Heilbronner, Rebecca Hoban, Liping Hou, Stéphane Jamain, Jean-Pierre Kahn, Layla Kassem, Tadafumi Kato, John R. Kelsoe, Sarah Kittel-Schneider, Sebastian Kliwicki, Po-Hsiu Kuo, Ichiro Kusumi, Gonzalo Laje, Catharina Lavebratt, Marion Leboyer, Susan G. Leckband, Carlos A. López Jaramillo, Mario Maj, Alain Malafosse, Lina Martinsson, Takuya Masui, Philip B. Mitchell, Frank Mondimore, Palmiero Monteleone, Audrey Nallet, Maria Neuner, Tomás Novák, Claire O’Donovan, Urban Ösby, Norio Ozaki, Roy H. Perlis, Andrea Pfennig, James B. Potash, Daniela Reich-Erkelenz, Andreas Reif, Eva Reininghaus, Sara Richardson, Guy A. Rouleau, Janusz K. Rybakowski, Martin Schalling, Peter R. Schofield, Oliver K. Schubert, Barbara Schweizer, Florian Seemüller, Maria Grigoroiu-Serbanescu, Giovanni Severino, Lisa R. Seymour, Claire Slaney, Jordan W. Smoller, Alessio Squassina, Thomas Stamm, Jo Steele, Pavla Stopkova, Sarah K. Tighe, Alfonso Tortorella, Gustavo Turecki, Naomi R. Wray, Adam Wright, Peter P. Zandi, David Zilles, Michael Bauer, Marcella Rietschel, Francis J. McMahon, Thomas G. Schulze, Martin Alda

**Affiliations:** 1 Department of Psychiatry, Dalhousie University, Halifax, Nova Scotia, Canada; 2 Department of Psychiatry and Psychotherapy, Charité Universitätsmedizin, Berlin, Germany; 3 Human Genetics Branch, Division of Intramural Research Programs, National Institute of Mental Health (NIMH), National Institutes of Health (NIH), Bethesda, Maryland, United States of America; 4 Unit of Clinical Pharmacology, University-Hospital of Cagliari, Cagliari, Italy; 5 Hôpitaux Universitaires de Genève, Department of Mental Health and Psychiatry, Geneva, Switzerland; 6 Department of Clinical Neuroscience, Karolinska Institutet, Stockholm, Sweden; 7 Department of Psychiatry, University of Campinas, Campinas, Brazil; 8 Department of Psychiatry, The University of Adelaide, Adelaide, Australia; 9 Assistance publique - Hôpitaux de Paris, Groupe Hospitalier Lariboisière-F. Widal, Pôle de Psychiatrie, Paris, France; 10 Department of Psychiatry, Medical University of Graz, Graz, Austria; 11 Department of Psychiatry, Mayo Clinic, Rochester, Minnesota, United States of America; 12 Division of Epidemiology and Genetics, Academia Sinica, Institute of Biomedical Sciences, Taipei, Taiwan; 13 Department of Genomics, Life and Brain Center and Institute of Human Genetics, Bonn University, Bonn, Germany; 14 Psychiatric Genetic Unit, Poznan University of Medical Sciences, Poznan, Poland; 15 Section of Neuroscience and Clinical Pharmacology, Department of Biomedical Science, University of Cagliari, Cagliari, Italy; 16 Department of Psychiatry and Behavioral Sciences, Johns Hopkins University School of Medicine, Baltimore, Maryland, United States of America; 17 Institut National de la Santé et de la Recherche Médicale, Unité 955, Institut Mondor de Recherche Biomédicale, Equipe 15, Faculté de médecine, Créteil, France; 18 Department of Psychiatry and Psychotherapy, Ludwig Maximilian University, Munich, Germany; 19 Neuroscience Research Australia - Genetics of Mental Illness and Brain Function, Sydney, Australia; 20 Service de psychiatrie, Hôpital Charles Perrens, Bordeaux, France; 21 Mood Disorders Center of Ottawa, Ottawa, Canada; 22 Department of Psychiatry, University of Toronto, Toronto, Canada; 23 Department of Psychiatry and Psychotherapy, Georg-August-Universität, Göttingen, Germany; 24 Osaka University Graduate School of Medicine, Osaka, Japan; 25 Psychiatric Genetic Unit, Poznan University of Medical Sciences, Poznan, Poland; 26 Department of Psychiatry, University of California San Diego, San Diego, California, United States of America; 27 Department of Psychiatry, Veterans Affairs San Diego Healthcare System, San Diego, California, United States of America; 28 Service de Psychiatrie et Psychologie Clinique, Centre Hospitalier Universitaire de Nancy, Nancy, France; 29 Laboratory for Molecular Dynamics of Mental Disorders, RIKEN Brain Science Institute, Saitama, Japan; 30 Department of Psychiatry, Psychosomatics, and Psychotherapy, University of Würzburg, Würzburg, Germany; 31 Department of Adult Psychiatry, Poznan University of Medical Sciences, Poznan, Poland; 32 Institute of Epidemiology and Preventive Medicine, National Taiwan University, Taipei, Taiwan; 33 Department of Psychiatry, Hokkaido University Graduate School of Medicine, Sapporo, Japan; 34 Department of Molecular Medicine and Surgery, Karolinska Institutet and Center for Molecular Medicine, Karolinska University Hospital, Stockholm, Sweden; 35 Department of Pharmacy, Veterans Affairs San Diego Healthcare System, San Diego, California, United States of America; 36 Skaggs School of Pharmacy and Pharmaceutical Sciences, University of California San Diego, San Diego, California, United States of America; 37 Department of Psychiatry, University of Antioquia, Medellin, Colombia; 38 Department of Psychiatry, University of Napoli, Napoli, Italy; 39 School of Psychiatry, University of New South Wales, and Black Dog Institute, Sydney, Australia; 40 Prague Psychiatric Center, University of Prague, Prague, Czech Republic; 41 Department of Psychiatry, Fujita Health University School of Medicine, Toyoake, Japan; 42 Department of Psychiatry, Nagoya University Graduate School of Medicine, Nagoya, Japan; 43 Department of Psychiatry, Massachusetts General Hospital and Harvard Medical School, Boston, Massachusetts, United States of America; 44 Department of Psychiatry and Psychotherapy, Technische Universität Dresden, Germany; 45 Department of Psychiatry, University of Iowa, Iowa City, Iowa, United States of America; 46 Centre of Excellence in Neuroscience of Université de Montréal, Centre Hospitalier de l’Université de Montréal and Department of Medicine, Université de Montréal, Montréal, Canada; 47 Alexandru Obregia Psychiatric Hospital, Biometric Psychiatric Genetics Research Unit, Bucharest, Romania; 48 McGill Group for Suicide Studies, Douglas Mental Health University Institute, Montréal, Canada; 49 The University of Queensland, Queensland Brain Institute, Brisbane, Australia; 50 Johns Hopkins Bloomberg School of Public Health, Department of Mental Health, Baltimore, Maryland, United States of America; 51 Department of Genetic Epidemiology in Psychiatry, Central Institute of Mental Health Mannheim, University Medical Center Mannheim, University of Heidelberg, Mannheim, Germany; Tokyo Metropolitan Institute of Medical Science, Japan

## Abstract

**Objective:**

The assessment of response to lithium maintenance treatment in bipolar disorder (BD) is complicated by variable length of treatment, unpredictable clinical course, and often inconsistent compliance. Prospective and retrospective methods of assessment of lithium response have been proposed in the literature. In this study we report the key phenotypic measures of the “Retrospective Criteria of Long-Term Treatment Response in Research Subjects with Bipolar Disorder” scale currently used in the Consortium on Lithium Genetics (ConLiGen) study.

**Materials and Methods:**

Twenty-nine ConLiGen sites took part in a two-stage case-vignette rating procedure to examine inter-rater agreement [Kappa (*κ*)] and reliability [intra-class correlation coefficient (ICC)] of lithium response. Annotated first-round vignettes and rating guidelines were circulated to expert research clinicians for training purposes between the two stages. Further, we analyzed the distributional properties of the treatment response scores available for 1,308 patients using mixture modeling.

**Results:**

Substantial and moderate agreement was shown across sites in the first and second sets of vignettes (*κ = *0.66 and *κ = *0.54, respectively), without significant improvement from training. However, definition of response using the A score as a quantitative trait and selecting cases with B criteria of 4 or less showed an improvement between the two stages (ICC_1_ = 0.71 and ICC_2_ = 0.75, respectively). Mixture modeling of score distribution indicated three subpopulations (full responders, partial responders, non responders).

**Conclusions:**

We identified two definitions of lithium response, one dichotomous and the other continuous, with moderate to substantial inter-rater agreement and reliability. Accurate phenotypic measurement of lithium response is crucial for the ongoing ConLiGen pharmacogenomic study.

## Introduction

Bipolar disorder (BD) is a lifelong and severe psychiatric illness characterized by recurrences of episodes of depression and hypomania/mania [1]. Lithium is among the first-line maintenance treatments for BD [2], [3], preventing relapses and recurrences of opposite polarity. In addition, lithium decreases the risk of suicidal behaviour and all-cause mortality in mood disorders [4]–[6].

Naturalistic analyses show that approximately one third of BD patients achieve complete remission on lithium [7]–[14]. Lithium-responsive BD patients have distinct clinical features, such as episodicity of clinical course [15], absence of rapid cycling [16], and a family history of BD [17], corresponding to the BD “core phenotype” [18].

Despite a significant genetic component for lithium-responsive BD [12], [19], pharmacogenetic studies have not produced replicated results [20], [21]. One possible explanation for the lack of conclusive pharmacogenetic findings is the varying definition of lithium response across the studies. Indeed, the assessment of lithium maintenance treatment response, and consequently the definition of the phenotype under study, is complicated by factors inherent to the natural history of BD. The irregular clinical course of BD [22] as well as variable treatment adherence [23] are only few of the factors that contribute to the complexity in assessing the response to lithium maintenance treatment.

To reduce the impact of the clinical heterogeneity of BD in pharmacogenetics (and possibly to define genetically more homogeneous subgroups of BD patients), researchers have proposed to select prospectively followed patients on lithium monotherapy with unequivocal clinical response [24], [25]. However, this may not be practical if large patient samples are needed. In such cases, we need to rely on retrospective evaluation of treatment response. Several such methods have been described in the literature including the Affective Morbidity Index (AMI) [26] and the Illness Severity Index [27]. The AMI takes into account the duration and the severity of an episode, the latter scored on a 4-point scale (0 = no conspicuous affective disturbance, 1 = mild depression or mania, 2 = moderate depression or mania, 3 = severe depression or mania). The area under the curve can be calculated from these two variables and compared between defined treatment periods. Similarly, the Illness Severity Index measures the efficacy of lithium treatment in controlling mood episodes. It is defined as the frequency of affective episodes prior to starting lithium adjusted for age at the time lithium was started [27]. However, changes of affective morbidity might be not only a result of the treatment, but could be due to other factors. In the Consortium on Lithium Genetics (ConLiGen, www.ConLiGen.org) study [28], we adopted the “Retrospective Criteria of Long-Term Treatment Response in Research Subjects with Bipolar Disorder” as the principal method of evaluation of the response to lithium [12], [13]. In addition to measuring the degree of clinical improvement, this scale weighs clinical factors considered relevant in determining whether the observed clinical change is in fact due to the lithium treatment.

Since ConLiGen is an international multi-centre collaboration, it has been crucial to assess the key phenotypic measures and the response to long-term lithium treatment reliability across the participating research groups. Here we present: 1) the results of the reliability analysis of response to lithium treatment across the participating centres, and 2) the distributional properties of the scale scores. These two sets of findings have been instrumental in obtaining stringent phenotypic definitions of lithium response. These analyses are of particular importance in light of the genome-wide association study (GWAS) currently being undertaken by ConLiGen.

## Materials and Methods

### Assessment of Clinical Response to Lithium Treatment

The response to lithium treatment was measured using a previously published and validated rating scale: the “Retrospective Criteria of Long-Term Treatment Response in Research Subjects with Bipolar Disorder” [12], [28]. Briefly, this scale quantifies the degree of improvement in the course of treatment (A criterion or A score) expressed as a composite measure of change in frequency and severity of mood symptoms. The A score is weighed against 5 factors (B criteria) which allow one to determine if the observed improvement is a result of the treatment rather than a spontaneous improvement or an effect of additional medication. Specifically, the B criteria consider: the number of episodes before/off the treatment (B1), the frequency of episodes before/off the treatment (B2), the duration of the treatment (B3), the compliance during period(s) of stability (B4) and the use of additional medication during the period of stability (B5). The total score (TS) is obtained by subtracting the B score from the A score.

### Analysis of the Inter-rater Agreement and Reliability of the Assessment of Lithium Response

The agreement and reliability of the assessment of lithium response between raters of 29 ConLiGen participating centres was measured using a two-stage case-vignette rating procedure (Table 1). Specifically, the study protocol had three phases: 1) twelve standardized case vignettes prepared by investigators (M.A., J.G., C.S.) at Dalhousie University were circulated and rated by 70 investigators; 2) annotated first-round vignettes and rating guidelines were circulated for training purposes after the first stage; 3) sixteen additional more complex vignettes prepared by senior researchers at Dalhousie University, Johns Hopkins University School of Medicine, National Institute of Mental Health (NIMH) and Academia Sinica of Taiwan (M.A., J.G., J.P., T.G.S., F.M., A.C.) were circulated and rated by 48 investigators at the participating sites. The first set of vignettes was based exclusively on BD patients who had been prospectively followed in a specialty program and with detailed clinical information on the course of illness and treatment history. The second set of vignettes was heterogeneous and included patients treated in various settings, some with limited clinical details assessed cross-sectionally. Since raters had no prior knowledge of the rating scale, this design allowed us to estimate the impact of training on agreement and reliability of lithium response assessment. The rating procedure was performed from April 2009 to October 2012.

**Table 1:** Number of raters from the Consortium on Lithium Genetics (ConLiGen) centres participating in the two-stage case-vignette rating procedure for inter-rater reliability and agreement.

ConLiGen centres	First stage	Second stage
University of Adelaide, Adelaide (Australia)	1	1
University of Sydney, Sydney (Australia)	1	0
University of Graz, Graz (Austria)	3	3
University of Campinas, São Paulo (Brasil)	3	3
Dalhousie University, Halifax (Canada)	9	2
University of Medellin, Medellin (Colombia)	4	4
Charles University, Prague (Czech Republic)	1	2
Institut national de la santé et de la recherche médicale, Paris (France)	1	1
University of Würzburg, Würzburg (Germany)	2	1
University of Göttingen, Göttingen (Germany)	2	0
Charité - Universitätsmedizin, Berlin (Germany)	1	2
Technische Universität Dresden,Dresden (Germany)	2	2
University of Cagliari, Sardinia (Italy)	3	3
University of Naples SUN, Naples (Italy)	1	2
The Japanese Collaborative Group on the Genetics of Lithium Response in Bipolar Disorder (Japan)*	4	4
University of Medical Sciences, Poznań (Poland)	2	2
Obregia Psychiatric Hospital, Medical University, Bucharest (Romania)	2	2
Karolinska Institutet, Stockholm (Sweden)	1	1
University of Geneva, Geneva (Switzerland)	3	2
Academia Sinica, Taipei (Taiwan)	1	1
National Taiwan University, Taipei (Taiwan)	2	2
National Institute of Mental Health (USA)	4	2
The Johns Hopkins University, Baltimore (USA)	7	5
Mayo Clinic, Rochester (USA)	6	1
Massachusetts General Hospital, Boston (USA)	2	0
University of California, San Diego (USA)	2	0
Total number of raters	70	48

ConLiGen: Consortium on Lithium Genetics.

* Hokkaido, Osaka, Tokio, Riken Brain Science Institute.

The degree of concordance of lithium response definition was assessed with Cohen’s kappa (*κ*) [29] and intra-class correlation (ICC) coefficient [30]. These analytical methods were applied to the dichotomous and continuous definition of lithium response, respectively. The *κ* statistics (multiple raters with two outcomes) were calculated with 95% confidence interval (CI) for each cut off point of the TS scale in the range from 3 (non response to lithium) to 8 (full response to lithium). Interpretation of the strength of agreement was made according to Landis and Koch: poor (*κ* <0.00), slight (0.00–0.20), fair (0.21–0.40), moderate (0.41–0.60), substantial (0.61–0.80), almost perfect (0.81–1.00) [31].

The quantitative scores of the treatment response scale were analyzed in the first (ICC_1_) and second (ICC_2_) stage of ratings. Specifically, we analyzed the TS (weighted clinical improvement), the A score (uncorrected clinical improvement), the B score (quantification of confounders), and the A score when B score ≤4. The latter measure allows the identification of “valid cases” through selection at the B criteria. Subjects with B score ≤4 are likely to have a clinical improvement causally related to lithium treatment. The ICC was tested with the two-way random effects model, that assumes a random sample of *K* investigators selected from a larger population, and each rates *N* targets (i.e., case vignettes) altogether, and the two-way mixed effects model, with each target rated by each of the same *K* investigators, who are the only ones of interest. For both models we calculated the single and average measure reliability.

### Analysis of the Distributional Properties of the Treatment Response Scale

For the analysis of the distributional properties, we accessed TS data of 1,308 BD patients from the NIMH centralized ConLiGen phenotypic dataset.

#### Mixture analysis: frequentist and Bayesian approach

We used mixture analysis to test whether we could identify subgroups of patients according to the degree of response to lithium as expressed by TS. The choice of the mixture model that best fit the distribution of TS was made according to the Akaike’s and Schwarz’s Bayesian information criteria (AIC and BIC, respectively). The lower values of these two criteria indicated the most parsimonious model that best fit the empirical function of total score distribution. The analysis was performed using the “NMixEM” function implemented in the MixAk package [32] of R software (version 2.13.2).

To verify the findings from the frequentist mixture analysis, we performed the Bayesian mixture analysis employing a minimum message length approach (MML) [33]. Specifically, we used the Snob software [34] to test whether the distribution results from a union of a number of “classes”, where the distributions “within-classes” are homogeneous and have a simple form, but vary significantly “between-classes”. The best fitting model was indicated as the most parsimonious model (i.e., the one with the lower cost expressed in nits, a specific measure unit conventionally used to express the length message). The analysis was performed using a measurement error equal to 2.5 empirically estimated by plotting the distribution of TS.

#### Cut off point calculation

Cut off points were derived using the theoretical TS function and calculating each data point’s probability of belonging to each class. Specifically, once the mixture model parameters were estimated, we calculated the posterior probability of any data point *x* belonging to the *i*-th class as
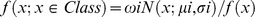
where *ω* is the weight, *μ* is the mean, *σ* is the standard deviation.

The resulting probabilities were then compared in order to establish which class the data point belonged to.

## Results

### Inter-rater Agreement and Reliability of the Assessment of Lithium Response

Raters agreed to a substantial/moderate (first stage of case-vignettes ratings) and moderate/fair (second stage of case-vignettes ratings) degree in assessing lithium response as a dichotomous variable (response/non response) (Table 2). We did not detect an effect of training as shown by the lack of improvement in *κ*. Specifically, in the first stage of ratings, the *κ* score showed a substantial level of agreement when we considered the TS cut off for response to lithium at 6 (*κ* = 0.65, 95% CI = 0.36–0.85) and at 8 (*κ* = 0.61, 95% CI = 0.33–0.83). The highest *κ* value was for the TS cut off point of 7 (*κ* = 0.66, 95% CI = 0.38–0.86). The second stage of ratings had overall lower *κ* values than the first indicating a moderate level of agreement in the assessment of lithium response (TS = 6: *κ* = 0.51, 95% CI = 0.29–0.73; TS = 7: *κ* = 0.54, 95% CI = 0.31–0.76; TS = 8: *κ* = 0.54, 95% CI = 0.28–0.76). Again, the highest κ value was found for the TS cut off point of 7. Details can be found in Table 2.

**Table 2:** Inter-rater agreement and reliability of the assessment of lithium response in the two-stage case-vignette rating procedure: kappa and intra-class correlation analysis.

Assessment of lithium response	First stage of ratings§				Second stage of ratings^∼^			
Dichotomous	*κ* (95% CI)	z		p	*κ* (95% CI)	z		p
TS cut off of 8	0.61 (0.33–0.83)	103.50		<0.00001	0.54 (0.28–0.76)	68.06		<0.00001
TS cut off of 7	0.66 (0.38–0.86)	112.18		<0.00001	0.54 (0.31–0.76)	68.71		<0.00001
TS cut off of 6	0.65 (0.36–0.85)	110.52		<0.00001	0.51 (0.29–0.73)	64.54		<0.00001
TS cut off of 5	0.58 (0.29–0.81)	99.23		<0.00001	0.48 (0.25–0.71)	61.25		<0.00001
TS cut off of 4	0.51 (0.20–0.78)	86.83		<0.00001	0.42 (0.18–0.67)	52.94		<0.00001
TS cut off of 3	0.40 (0.10–0.73)	68.25		<0.00001	0.37 (0.13–0.66)	47.46		<0.00001
**Continuous**	**ICC_1_ single measure** **(95% CI)** *		**ICC_1_ average measure** **(95% CI)** *		**ICC_2_ single measure** **(95% CI)** *		**ICC_2_ average measure** **(95% CI)** *	
TS	0.74 (0.59–0.89)		0.99 (0.98–1.00)		0.55 (0.36–0.80)		0.98 (0.96–0.99)	
A score	0.66 (0.49–0.85)		0.99 (0.98–1.00)		0.52 (0.33–0.78)		0.98 (0.96–0.99)	
Total B score	0.59 (0.41–0.81)		0.99 (0.98–1.00)		0.34 (0.19–0.64)		0.96 (0.92–0.99)	
A score if total B score ≤4	0.71 (0.51–0.91)		0.99 (0.99–1.00)		0.75 (0.51–0.96)		0.99 (0.98–1.00)	

TS: total score.

ICC: intra-class correlation.

CI: confidence interval.

* Mixed and random effects models.

§ 70 raters.

¶ 48 raters.

We then analyzed the inter-rater reliability for the continuous definition of lithium response. We found that ICC values (two-way random and mixed effects models, single measure) were higher in the first stage of ratings for TS (ICC_1_ = 0.74 versus ICC_2_ = 0.55), for A score (ICC_1_ = 0.66 versus ICC_2_ = 0.52) and for total B score (ICC_1_ = 0.59 versus ICC_2_ = 0.34). However, the training improved the inter-rater reliability of the A score when B score was ≤4 (ICC_1_ = 0.71 versus ICC_2_ = 0.75). These results are outlined in Table 2.

### Analysis of the Distributional Properties of the Treatment Response Scale

#### Distribution of the TS and joint distribution with score A

**Figure 1:**
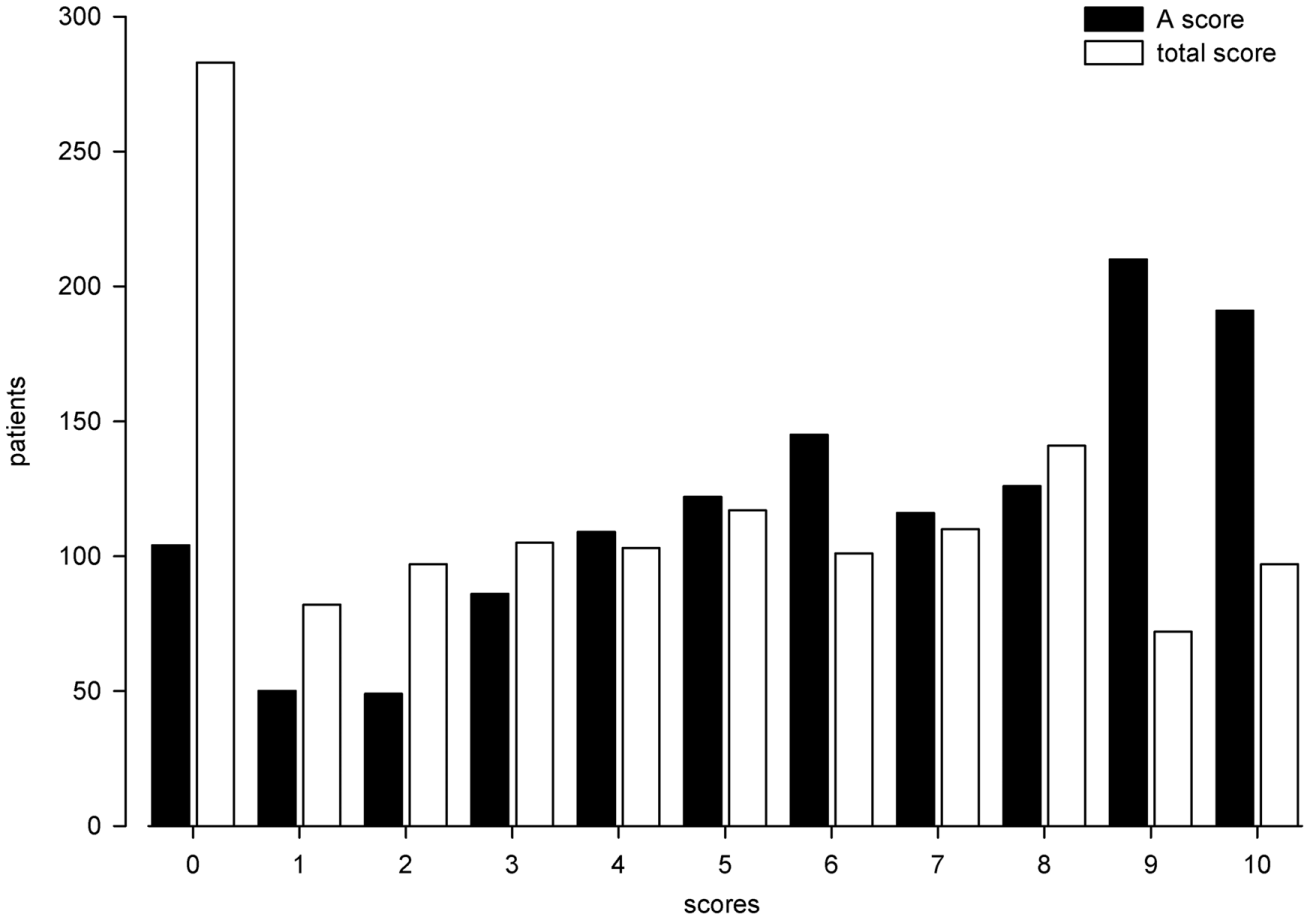
Distribution of total and A scores in the Consortium on Lithium Genetics sample. Histogram plot of the scale scores in 1,308 bipolar disorder patients characterized for response to lithium maintenance treatment.

**Figure 2:**
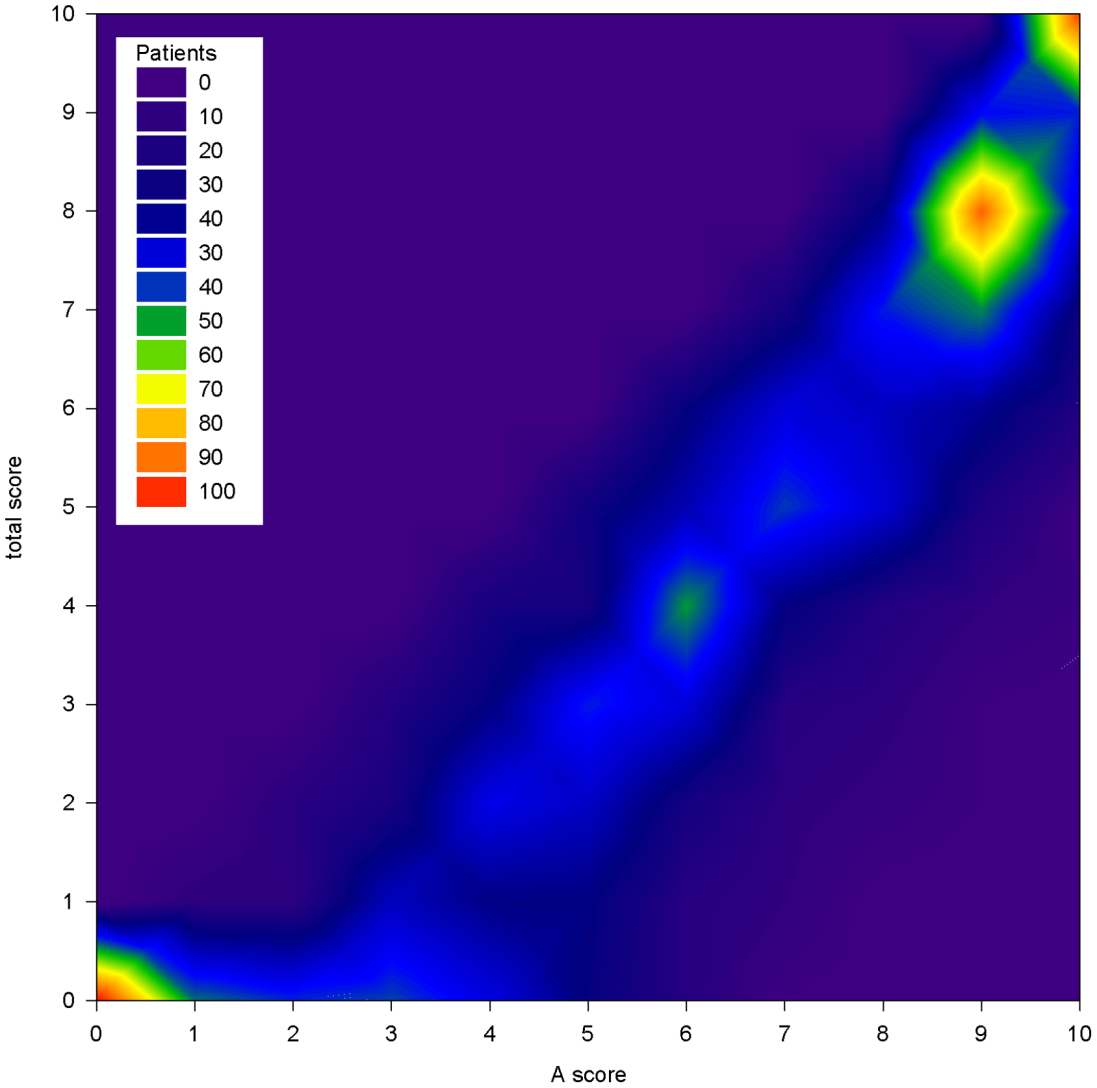
Joint distribution of total and A score in the Consortium on Lithium Genetics sample. Contour plot of the scale scores in 1,308 bipolar disorder patients characterized for response to lithium maintenance treatment.

Figure 1 illustrates the distribution of TS and A score in 1,308 BD patients characterized for lithium response. Two hundred eighty three patients (21.6%) had TS equal to 0 and 104 patients (8%) had A score equal to 0. In the whole sample the mean A score ± standard deviation] was 6.1±3.1 and the mean TS was 4.4±3.1. The joint distribution of TS and A scores is represented in Figure 2. It illustrates the presence of two frequency peaks at the extreme ends of the scale, namely at 0 and in the area comprised between score A equal to 9 and TS equal to 8–10. A third peak is present at the intersection of A score equal to 6 and TS of 4.

#### Mixture analysis: frequentist and bayesian approach

**Figure 3:**
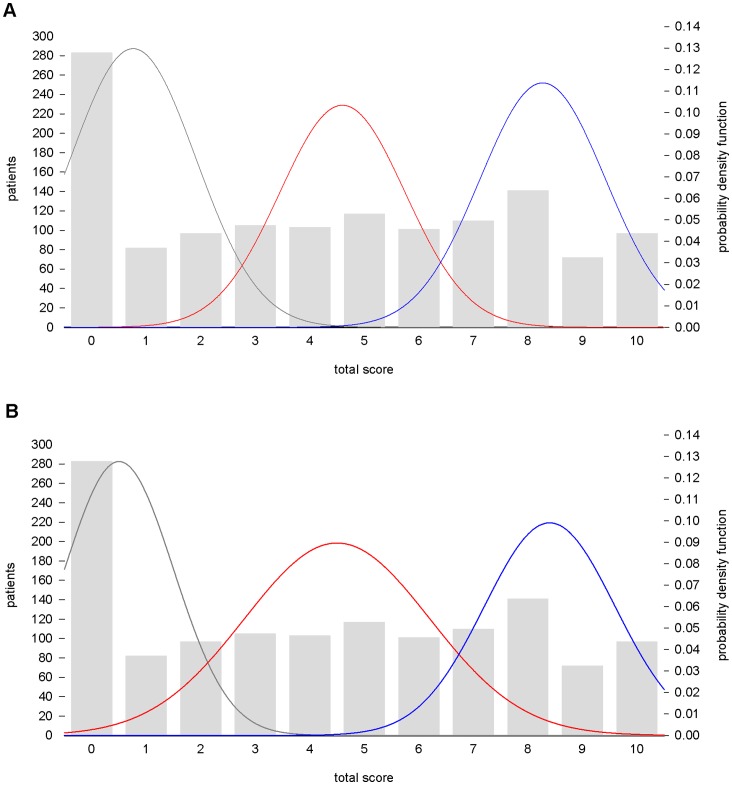
Empirical and theoretical distributions of the total score in the Consortium on Lithium Genetics sample. Frequentist, **A**, and Bayesian minimum message length, **B**, mixture modeling identify three subpopulations of non responders (grey), partial responders (red), and full responders (blue) in total scores of 1,308 bipolar disorder patients characterized for response to lithium maintenance treatment.

The frequentist mixture analysis on TS showed a best-fitting theoretical model of three normal components (AIC = 6467.69, BIC: 6498.75) (Figure 3A). A model with four components did not improve the fit (AIC = 6471.68, BIC = 6513.09, respectively). The mean TS was 0.76±1.15 for the non responder component, 4.6±1.15 for the partial responder component and 8.3±1.15 for the full responder component, with 37%, 30%, and 33% of the population proportion, respectively.

The MML mixture analysis identified the most parsimonious model of three normal components [mean, (SD), (proportion of population)]: 0.5, (1.00), (32%); 4.5, (1.7), (38%); 8.4, (1.2), (30%)], representing the non responder, the partial and the full responder groups of patients. The model is displayed in Figure 3B.

#### Cut off point calculation

The functions of TS identified with the two different mixture analysis approaches (frequentist and Bayesian) were used to derive the probability of belonging and to calculate the cut off point between the components. The frequentist mixture model suggested two cut off points at TS = 3 and TS = 6.4. Considering the Bayesian MML theoretical function, we obtained two cut off points at 2 and 7. These results confirmed that TS ≥7 is the most appropriate cut off for the definition of full response to lithium prophylaxis as suggested in previous studies [12], [13].

## Discussion

The purpose of this study was to assess the key phenotypic measures of response to lithium treatment in the large international collaborative Consortium on Lithium Genetics. To this end, two main analyses have been carried out: the inter-rater agreement and reliability of lithium response definition across the ConLiGen participating sites, and the analysis of the distributional properties of the lithium treatment response scale [12]. We found that two definitions of lithium response, one dichotomous and the other continuous had moderate to substantial inter-rater agreement and reliability. Specifically, the two-stage case vignettes inter-rater reliability analysis pointed to the measure of clinical improvement under lithium treatment expressed by the A score and with selection of “valid cases” through a total B score ≤4. This phenotypic definition of lithium response had a substantial inter-rater reliability in the first stage of ratings (ICC_1_ = 0.71) with further improvement in the second stage (ICC_2_ = 0.75).

Regarding the dichotomous definition of lithium response, a scale TS ≥7 was identified as the best cut off as shown by inter-rater agreement *κ* scores in the first (*κ* = 0.66) and second (*κ* = 0.54) stages of case vignette ratings. Further, the analysis of the distributional properties of the treatment response scale further supported this dichotomous definition. In addition, this same measure of lithium response has been previously proposed in several clinical and genetic papers [12], [13], [35], [36].

Some methodological considerations need to be made. For the analysis of the distributional properties, we applied mixture modeling, a method that has been extensively used in psychiatry for the identification of patient subgroups, reducing phenotypic heterogeneity and ultimately helping genetic research [37]–[39]. It should be noted that this method is exploratory and it does not identify the factors determining the differences between the identified subgroups [40]. A validation of the model can be obtained by comparison of the characteristics of each subgroup. In the ConLiGen study, we plan to use the clinical correlates of lithium response as external validators of the phenotypic measure suggested by the mixture modeling. Such analysis will test and compare the direction and magnitude of the association of a number of clinical variables with lithium response in its dichotomous and continuous definition.

Notably, the analysis of inter-rater reliability and agreement has involved investigators belonging to different research groups with different clinical backgrounds and training. Nevertheless, the use of standardized case vignettes and the training procedures has produced moderate to substantial agreement in the assessment of lithium response. These findings are of importance, given the evidence that even in the context of inpatient unit settings the inter-rater agreement can be unsatisfactory [41].

We performed a two-stage case-vignettes procedure aimed at testing the effect of training on the assessment of lithium response. Contrary to our expectations, we only detected improvement in the inter-rater reliability of lithium response expressed by the A score and with selection of “valid cases” through a total B score ≤4, but not in that expressed by TS or A score. Arguably, the second set of vignettes described more complicated clinical cases with comorbidities, lack of compliance and multiple treatments, all factors that could have influenced the scoring of the B criteria. Indeed, the ICC for the total B score decreased noticeably in the second stage of ratings, implying an increased variability in rating that impacted the discrimination among cases [42]. This explanation is corroborated by the finding of the higher ICC_2_ of A score with total B score ≤4. By applying this cut-off we decreased the assessment variability ultimately increasing the discrimination among cases.

Further, these findings confirm that patients with short duration of lithium treatment, poor compliance, and concomitant medications are unlikely to be assessed reliably. This argues against the inclusion of such complex, non-standard cases in pharmacogenomic studies of lithium response. Finally, the higher inter-rater agreement and reliability found in the first set of vignettes suggests that the assessment of lithium response is reliable if sufficient clinical details are available. On the other hand if the information is limited, additional rater training will be of little help.

In conclusion, our findings support the use of two definitions of lithium response for the pharmacogenomic GWAS currently being performed by ConLiGen. Accurate phenotypic definitions of treatment response are crucial in pharmacogenomic studies [43], [44]. Heterogeneity in the phenotype definition of treatment response can be a problem especially when in the context of psychiatric disorders. In the absence of other reliable clinical measures of response to lithium, this study has suggested two plausible phenotypic definitions that await application and validation in other samples.
